# An Analysis of Diet Quality, How It Controls Fatty Acid Profiles, Isotope Signatures and Stoichiometry in the Malaria Mosquito *Anopheles arabiensis*

**DOI:** 10.1371/journal.pone.0045222

**Published:** 2012-10-25

**Authors:** Rebecca Hood-Nowotny, Bettina Schwarzinger, Clemens Schwarzinger, Sharon Soliban, Odessa Madakacherry, Martina Aigner, Margarete Watzka, Jeremie Gilles

**Affiliations:** 1 Department of Terrestrial Ecosystem Research, University of Vienna, Vienna, Austria; 2 Insitute for Chemical Technology of Organic Materials, Johannes Kepler University Linz, Linz, Austria; 3 Insect Pest Control Laboratory, Agency’s Laboratories Seibersdorf, Joint Food and Agriculture Organization/International Atomic Energy Agency Division of Nuclear Techniques in Food and Agriculture, International Atomic Energy Agency, Vienna, Austria; New Mexico State University, United States of America

## Abstract

**Background:**

Knowing the underlying mechanisms of mosquito ecology will ensure effective vector management and contribute to the overall goal of malaria control. Mosquito populations show a high degree of population plasticity in response to environmental variability. However, the principle factors controlling population size and fecundity are for the most part unknown. Larval habitat and diet play a crucial role in subsequent mosquito fitness. Developing the most competitive insects for sterile insect technique programmes requires a “production” orientated perspective, to deduce the most effective larval diet formulation; the information gained from this process offers us some insight into the mechanisms and processes taking place in natural native mosquito habitats.

**Methodology/Principal Findings:**

Fatty acid profiles and *de-novo* or direct assimilation pathways, of whole-individual mosquitoes reared on a range of larval diets were determined using pyrolysis gas chromatograph/mass spectrometry. We used elemental analysis and isotope ratio mass spectrometry to measure individual-whole-body carbon, nitrogen and phosphorous values and to assess the impact of dietary quality on subsequent population stoichiometry, size, quality and isotopic signature. Diet had the greatest impact on fatty acid (FA) profiles of the mosquitoes, which exhibited a high degree of dietary routing, characteristic of generalist feeders. *De-novo* synthesis of a number of important FAs was observed. Mosquito C:N stoichiometry was fixed in the teneral stage. Dietary N content had significant influence on mosquito size, and P was shown to be a flexible pool which limited overall population size.

**Conclusions/Significance:**

Direct routing of FAs was evident but there was ubiquitous *de-novo* synthesis suggesting mosquito larvae are competent generalist feeders capable of survival on diet with varying characteristics. It was concluded that nitrogen availability in the larval diet controlled teneral mosquito size and that teneral ***C***N ratio is a sex- and species-specific fixed parameter. This finding has significant implications for overall mosquito competitiveness and environmental management.

## Introduction

Understanding mosquito ecology has recently been prioritized as a prerequisite for malaria eradication [1]. Ferguson *et al.* stressed that our knowledge of mosquito ecology is minimal compared to that of other agricultural pests and model organisms, and suggested the reasons for this are institutional compartmentalization and cultural effects, research having focused on medical issues, largely overlooking the mechanisms and ecology of vector transmission.

As mosquito vectors are embedded within ecological communities as predators, prey and competitors, an understanding of their ecology is essential to avoid any interventions triggering cascades of ecological effects that could lead to enhanced malaria transmission [1]. With over thirty different primary vectors dominating transmission, an understanding of the competitive interactions and species specific niche adaptations is critical for effective vector management. Although many studies have shown that the growth rates of larval mosquito vectors are negatively correlated with their population size, resulting in smaller, more robust and fecund populations, the mechanisms underlying this plasticity are largely unexplored [1], [2], [3]. Body size has also been shown to have important fitness implications, however individual body size frequency distributions within a population remain under-investigated in insects in general [4]. One of the key factors controlling population dynamics and body size is larval nutrition, and previous studies have shown that nitrogen (N) and phosphorus (P) availabilities are important ecological determinants in other insects [3], [5]. However, it is extremely difficult to study nutritional impacts on such small insects and generally methods of analysis are laborious and complex, often limiting the scope of the studies conducted. Here we present some rapid techniques that may overcome some of these constraints opening up opportunities for more holistic ecosystem based research.

Advances in elemental analysis and pyrolysis techniques to measure fatty acid concentrations, mean that it is now possible to investigate nutritional impacts on mosquito larvae development and survival on an individual basis. This allows us to explore mosquito larval development within the larger ecological framework and relate it to current paradigms in ecological thinking, such as ecological stoichiometry. Ecological stoichiometry has been heralded as the unifying theory of ecology. It is based on simple laws of physics such as mass balance and energy dissipation meshed with the biological principles of energy tradeoffs at biochemical and individual levels. These principles have been cleverly honed to explain the dynamics of individuals, populations, communities and ecosystems [6].

At the very base of ecological stoichiometry theory is the concept that at the organism level there is a unique balance of multiple chemical substances, mainly ratios of carbon:nitrogen:phosphorus (C:N:P) and the consequence of this homeostasis is that nutrient cycles and processes at higher scales in the ecosystem are driven. Fundamentally the theory suggests that living organisms are constrained and different from their environment, and in almost all circumstances will be limited by one element; usually but not exclusively, nitrogen or phosphorous [5], [6]. Although this is a universal phenomenon, little is known of the extent to which stoichiometry drives population dynamics and its consequences for general mosquito biology. Stoichiometric theory contrasts to the current theory that mosquito larval nutrition is a complex combination of dietary requirements. In this study we set out to test whether these theories hold up for *Anopheles arabiensis* mosquitoes and whether they might explain some observed phenomenon of population plasticity [7].

Mosquito larval nutrition has been extensively studied; it is known that proteins (or amino acids), sugar (glucose or sucrose), polyunsaturated fatty acids (PUFAs), sterols, vitamins and nucleotides are all essential for mosquito development. It has been shown that at least fourteen amino acids are essential for larval growth and survival [8], [9], [10], [11]. Additionally, minimal concentrations of essential vitamins are required to ensure optimal growth of several mosquito species [12], [13]. Dadd & Kleinjan [13] also showed that less that 5% of *Cx. pipiens* larvae reached adult stage in diets lacking a combination of three nucleotides, demonstrating their role in nutrition. It is well documented that mosquito larval diet quality and quantity influences both adult quality and in turn sexual competitiveness [7], [8], [14]. Efficient and economic mass rearing of any insect requires an in-depth understanding of the dietary components which influence insect quality [15]. A broad understanding of dietary requirements and influences can also yield an interesting insight into the natural ecology and biology of the insect. For example, laboratory experiments have shown that supplementary protein feeding of fruit flies led to more successful mating behavior, a critical issue in both insect ecology and sterile insect technique programmes [16].

The stoichiometric paradigm suggests that many of the reductionist investigations that determine the specific chemical requirements for the successful nutrition of an organism, overlook the ubiquitous presence of the individual components in the ecosystem as a whole. It suggests in fact that systems are generally constrained by specific macro-nutritional requirements which have the individual components embedded within them, and that primary producers and thus secondary and higher level consumers are ultimately constrained by biogeochemistry. It is well documented that primary producers respond positively to inputs of nitrogen and phosphorous, as these are the limiting elements in most natural systems [17]. Extending from this, evolutionary theory states that the fittest individuals will use the available resources for reproduction most efficiently and therefore their genes will dominate. Evolutionary logic would suggest that generalist secondary consumers would thus adapt to utilize the most commonly present components in primary producers and that these would be used in the most energy efficient manner [18], [19]. Indeed current trophic interaction research suggests that it is energetically more efficient to incorporate dietary fatty acids (FAs) directly into the consumer’s tissue without degradation or modification, a process termed dietary routing [20].

Fatty acid profiles provide a large amount of information on the development, reproduction, health and feeding ecology of organisms [21], [22]. Previous studies have stressed that mosquitoes are unable to elongate the 18C poly unsaturated FAs and thus C18, C20 and C22 polyunsaturated fatty acids are essential for larval development, adult survival and flight [23]. However in natural aquatic environments these fatty acids may be present and are possibly not limiting constraints of larval nutrition *per se* [24].

In most ecological systems energy transfer and energy flux is thought to be the primary constraint on total ecosystem productivity [25]. Here we present a framework with which we test the hypothesis that dietary quality, or nutrient content, i.e. stoichiometry, plays a significant role in regulating ecosystem energy and nutrient transfers in primary consumers and can be used as a predictive tool of population response.

Studying larval nutrition within the context of developing diets for mass reared anopheles mosquitoes gave us the opportunity to test a number of hypotheses that would support or discredit the stoichiometry theory. The pyrolysis GCMS system offered us the chance to engage in a simple comprehensive analysis of the fatty acids in the diet and their effects on individual mosquito FA profiles. Elemental analysis and isotope ratio mass spectrometry of whole mosquitoes meant that we could compare whole body carbon, nitrogen and phosphorous and macro nutrient levels, in addition to the fatty acid profile of the diet, giving us insight into the important features of diet composition. Finally, the isotope analysis enabled us to investigate some of the current assumptions in isotope ecology and test those assumptions in controlled environments, allowing us to confidently apply these techniques in future field studies [26]. Central null hypotheses we set out to explore were:

1. Fatty acid composition of mosquitoes is fixed and not influenced by the diet.
2. Mosquito stoichiometry is fixed and not influenced by the larval diet.

## Materials and Methods

### Mosquito Stocks and Rearing Methods

All experiments were conducted at the Insect Pest Control Laboratory (Joint FAO/IAEA Division) in Seibersdorf, Austria, in climate-controlled rooms maintained at 27°C ±1°C and 60% RH ±10%, with LD 12∶12 h photoperiod, including dusk (1 h) and dawn (1 h).

A stock strain of *Anopheles arabiensis* MRA-856 (available from MR4, MRA-856), was used in all the experiments. Having originated from Dongola, Northern Sudan (2005) the strain has since been maintained on a Koi Floating Blend® diet for approximately 105 generations.

Eggs were hatched at a low density.

### Experimental Designs

#### Experiment 1. Initial investigations of TBN, TBC, C:N ratios and wing length

This experiment set out to investigate the relationship between total body carbon (TBC) and nitrogen (TBN) and wing length, thus a range of diet concentrations were fed to the mosquitoes to get a range of mosquito size classes, but basically fed on the same diet. Less than 4 hours after eclosion, 32 larvae (1^st^ instar) were transferred into six 9 cm diameter Petri dishes containing 32 ml of deionised water [7] and these were fed daily with 2 ml of a 1, 1,5, or 2% solution of a KD1 diet which was a mixture of ground wheat, corn, bean, chick pea, rice, bovine liver powder (BLP) and Vita mix in the following ratio: 2∶2:2∶2:2∶2:2.6. Six replicate dishes were set up for each treatment. All pupae were collected daily and live un-fed teneral adults collected within 12 hours. Wing length was determined for each specimen: briefly, a wing was clipped and mounted on a slide, and a digital image taken using a camera mounted on a stereo microscope (CC-12 camera, Olympus Soft Imaging Solutions). Wing length was measured from the alula notch to the wing tip; measurements were performed with AnalySIS FIVE software (Olympus Soft Imaging Solutions) [27]. Wings were re-united with their bodies in the tin cups used for analysis and thus whole adult mosquitoes could be analysed for total body carbon and nitrogen and their isotopic ratios as described below. This meant TBC and TBN could be compared against wing length at the level of individual mosquitoes.

#### Experiment 2. Feeding experiments for In-depth Dietary analysis

In this experiment we set out to determine which factors of nutritional quality could influence mosquito size and whether fatty acids were directly routed from diet to the consuming larva and consequently preserved in the adult mosquito.

The sixteen diets tested in this experiments were AP100 (Zeilgler USA, a commercially available shrimp larval diet), bean powder, bovine liver powder (BLP), brewer’s yeast, carrot, chick pea, corn, rice, soy hydrolysate, spirulina, squid liver powder (SLP), tuna, vitamin-mix, wheat, wheat bran and yeast hydrolysate.

Five hundred larvae (L1 instar) were counted into a tray (30×40 cm) containing 1.5 L of de-ionized water. Due to the sensitivities of anopheline species to overfeeding and larval habitat fouling, the mosquitoes were fed on demand (approx. 0.25 mg/larva/day). This diet was added in a ground form in quantities aiming to attain maximum adult survival based on the colour and state of the larval water and the previous experience of the technicians. Newly formed pupae were transferred to emergence tubes. Upon adult emergence, ten males and ten females were transferred to Eppendorf tubes and frozen. Care was taken to sample the first ten males and first ten females that emerged from each treatment to overcome any emergence date bias. These were randomly divided into three batches and triplicate whole mosquito samples of each sex were analysed for fatty acids (Py-GCMS), TBN and TBC and their respective ^15^N and ^13^C values (Elemental-IRMS), and TBP (Total body phosphorous).

#### Experiment 3. Determining the influence of dietary N and P on mosquito survival and production

This experiment was set up to determine the influence of dietary N and P concentration on adult and pupal survival. Triplicate sets of 16 1^st^ instar larvae were loaded into 35 mm diameter petri dishes containing 16 ml of water. Each dish daily received 1 ml of a 1% solution of one the sixteen different larval foods listed above. It has been shown in previous experiments that a 1% concentration was the concentration where sufficient food was available but was least likely to produce water fouling and associated effect on the population size. Pupation date was noted and adults collected as described above.

### Sample Analysis

#### Pyrolysis GCMS for fatty acid analysis

Typically 100 µg of diet or a complete mosquito specimen were put into a quartz tube and 4 µl of a diluted, aqueous solution of tetramethylammonium hydroxide (TMAH) was added. The samples were subsequently pyrolyzed at 450°C for 10 s with a CDS 5250 pyrolysis autosampler attached to a Thermo Trace GC Ultra/MD 800 gas chromatography/mass spectrometry system. Volatile products were separated on a Supelco SP 2330 column (30 m, ID 0.32 mm, 0.2 µm film thickness) with helium 4.6 as carrier gas (2 ml.min^−1^) and identified by interpretation of their EI mass spectra and comparison to NIST 2002, Wiley, and NBS electronic libraries. The pyrolysis interface was kept at 300°C, the GC/MS interface at 280°C; the GC was programmed from 100°C (1 min) to 230°C (5 min) at a rate of 10°C min^−1^. The mass spectrometer was operated in EI mode (70 eV) at a source temperature of 200°C. The method was optimised based on the standard linseed oil, a triglyceride based oil, with several unsaturated fatty acids. An optimal thermally assisted hydrolysis and methylation method was developed to avoid the known problems of isomerisation.

Py-GCMS requires no sample preparation apart from the addition of TMAH. Analysis typically takes 20 minutes per sample and 100 µg C is the ideal sample size. In contrast fatty acids are conventionally measured by gas-liquid chromatography (GLC) using a flame ionisation detector, following a complex procedure of lipid extraction, purification, transesterification and methylation of approximately 50–70 mg of sample. The sample preparation procedure typically takes 2–3 days.

#### Elemental and stable isotope analysis

Whole single mosquito samples and ground diet samples were dried at 60°C for 24 h, placed into 8 by 5 mm tin cups and analyzed at SILVER, Vienna University for total N, C,^15^N and ^13^C, using an isotope ratio mass spectrometer (Delta PLUS, Thermo Finnigan, Germany) interfaced with an elemental analyzer (Flash EA, CE Instruments, UK). Samples were combusted in an atmosphere of oxygen at 1,020°C and passed over chromium oxide and silvered cobalt oxide for complete oxidation, and subsequently over hot copper (640°C) to reduce oxides of nitrogen to elemental nitrogen (N_2_). The resultant gas was carried in a stream of helium through a scrubber to remove residual water and was then passed over a gas chromatographic column to separate N_2_ and CO_2._ Peaks were bled into the mass spectrometer to determine the isotopic ratios. A full complement of internal and external standards was run with the samples to calculate isotopic ratios, % N and % C values. The isotope ratios were expressed as parts per thousand per mille (‰) or δ deviation from the internationally recognized standards, Vienna Pee Dee Belemnite (VPDB) and atmospheric nitrogen [28]. ^15^N makes up 0.3663% of all N at atoms natural abundance levels in air, the delta notation is basically deviation from this value multiplied by a thousand. Similarly the deviation from VPDB multiplied by a thousand gives the delta notation for ^13^C.

### Phosphorous Analysis

Phosphorous analysis of whole mosquitoes or ground diet samples was conducted based on a wet digestion [29]. Dried single whole mosquitoes or 3–5 mg of diet (noted to nearest µg) were placed into individual 10 ml test tubes and 1.0 ml of 98% H_2_SO_4_ added. The tubes were heated in an aluminium heating block until they reached a temperature of 150°C, at which point 0.75 ml of 30% H_2_O_2_ was added drop by drop until the solid disappeared, excess H_2_O_2_ was boiled off at 150°C, samples cooled and their volume noted. Aliquots (0.5 ml) of sample were diluted 1∶10 with deionised distilled H_2_O and brought to pH 5 with NaOH, determined using a phenolphthalein indicator, and made up to a known volume. Samples were analysed using the microtitre plate Malachite green method [30]. 200 µl of sample or potassium di-hydrogen phosphate (KH_2_PO_4_) standard was mixed with 40 µL of Reagent 1 (14.2 mmol L^−1^ ammonium molybdate tetrahydrate in 3.1 *M* H_2_SO_4_) and shaken for 10 minutes. Following shaking 40 µL of Reagent 2 was added and the plate again shaken for a further 20 minutes before being read at 630 nm on a Tecan, micro-titre plate reader. Reagent 2 was prepared by adding 3.5 g L^−1^ aqueous polyvinyl alcohol (PVA) reagent (molecular weight between 31 000 and 50 000) to 500 ml of 80°C deionised distilled water and stirring it, after cooling to room temperature, 0.35 g of Malachite Green oxalate (Merck, Art. No. 1398) was added and made up to 1 litre using distilled deionised water. All values were compared to standards and calculations done to give µg P per mosquito or % P of the diet.

### Statistical Analysis

Statistical analyses were performed using Microsoft Excel, Statgraphics Plus, Centurian USA and Primer 6 version 6.18. software. In all cases, the significant alpha level was taken as *P*<0.05.

## Results

### Experiment 1. Initial Exploratory Experiment to Investigate the Relationship between Total Body Carbon and Nitrogen and Mosquito Wing Length

**Figure 1:**
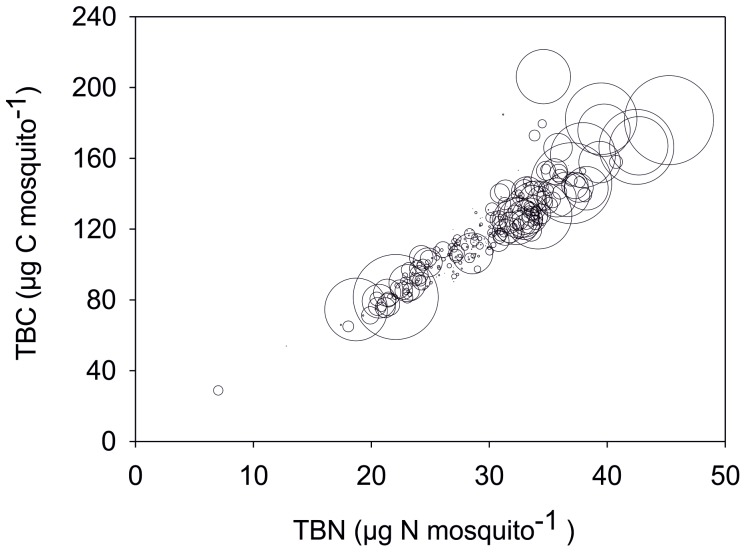
Experiment 1. Total body nitrogen (TBN) versus total body carbon (TBC) of individual *Anopheles arabiensis* mosquitoes, where bubble size represents the square of the deviation from average wing length.

Simple regression and multiple regression analyses were used to determine the interactions, well aware of the possible interdependence of some of the variables. There was a weak but significant correlation between total body carbon (TBC) and wing length (r^2^ = 0.47 p<0.0001). However, there was a stronger correlation for total body nitrogen (TBN) and wing length (r^2^ = 0.61 p<0.0001), and this interaction was stronger in females than males. In addition, deviation from average wing length appeared to be nitrogen (N) dependent (Table 1, Figure 1). There was a strong correlation between TBN and TBC (r^2^ = 0.84 p<0.0001, Figure 1). In addition mosquito C:N ratio correlated weakly but significantly with wing length (r^2^ = 0.01 p<0.036) and more strongly with the independent variable δ^13^C (r^2^ = 0.49 p<0.0000); there was no correlation with δ ^15^N. This latter point could be explained by the fact that mosquitoes with greater N and subsequently greater C accumulation underwent enhanced lipogenesis which has been shown to be initiated in fruit flies fed N-rich diets [31]. This theory is supported to some extent by the non-independent relationship between C:N ratio and TBC, which was best described by a polynomial function (r^2^ = 0.48 p<0.000) rather than a direct linear function, which would suggest lipogenesis above a threshold N value. Lipids are known to be depleted in ^13^C in relation to bulk tissues [32] due to isotopic discrimination by key enzymes. In short this indicates that beyond a certain N threshold mosquitoes just got fatter rather than bigger.

**Table 1:** Regression analysis of Experiment 1 data, (linear unless otherwise indicated).

	F	Df	p-Value	R squared
Against wing-length				
Female µg N	244	139	0.0000	0.63
Male µg N	89	147	0.0000	0.37
*Both*	449	286	0.0000	0.61
Female µg C	151	139	0.0000	0.52
Male µg C	90	147	0.0000	0.38
*Both*	258	286	0.0000	0.47
*C:N ratio (both)*	4	286	0.0360	0.01
TBN (µg N) vs TBC (µg C)	83	286	0.0000	0.84
C:N ratio vs δ ^13^ C	278	286	0.0000	0.49
C:N ratio vs δ^ 15^N	NS			
Multiple regression (TBN&TBC vs WL)	227	286	0.0000	0.61
Polynomial regression C:N vs TBC (µg C)	133	285	0.0000	0.48

NS denotes not significant at p>0.05 level.

Although TBN and TBC increased linearly with emergence date, the differences between sampling dates, as determined by ANOVA, were small but significant (p = 0.0009 for TBC and P = 0.0235 for TBN). Approximately 1% of the mosquitoes emerged on the initial emergence day followed by 44% on day two, with 38, 6 and 3% emerging on the following consecutive days. Thus the slight increase in TBN and TBC could be explained by the lack of competition for resources.

### Experiment 2. Feeding Experiments for In-depth Dietary Analysis

The pyrolysis method gave similar patterns of relative fatty acid composition of the three diets BLP, SLP and Tuna compared to the conventional method [33], revealed using simple regression analysis (r^2^ = 0.816, 0.726, 0.778 for the three diets, respectively) as has been previously demonstrated [34]. It was impossible to compare measurements of single mosquitoes using the conventional methods due to the constraints described in the methods section, so based on these results, previous conclusions [34], and the appropriate analysis of standards it was assumed that the method was suitable for the analysis of FAs in single mosquitoes.

**Figure 2:**
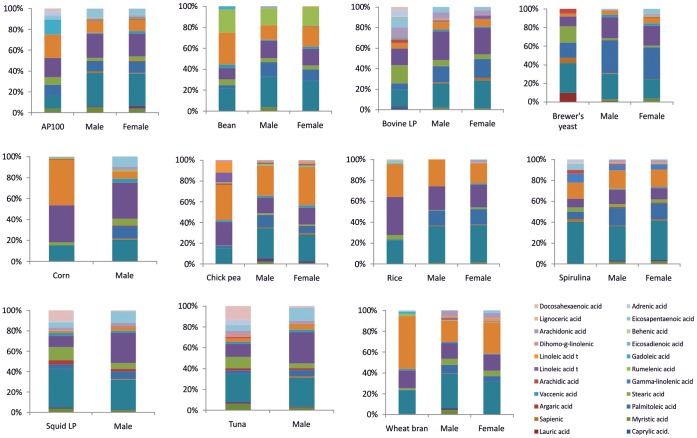
Experiment 2. Average relative fatty acid composition of diets and *Anopheles arabiensis* mosquitoes reared on the different diets (typically n = 3).

The wheat spectra were unusual in that only one fatty acid (palmitic acid 16∶0) appeared to be present, compared to published data in which palmitic 16∶0, stearic (18∶0), behenic (22∶0) oleic (18∶1) and linoleic (18∶2) acids were measured in significant concentrations in wheat flour [35], [30], therefore this sample was excluded from the fatty acid analysis. On some diets, notably the carrot, yeast hydrolysate, bean, vitamin mix and soy hydrolysate, mosquitoes failed to grow to adulthood and so these were also excluded from the analysis (Table 2, Figure 2.).

**Table 2:** Average relative fatty acid composition of diets and mosquitoes (no of replicates given as n in parenthesis).

Diet fatty acid	Isomer	AP100	Bean	BLP	Brewerśs Yeast	Chick Pea	Corn	Rice	Spirulina	SLP	Tuna meal	Wheat Bran							
Caprylic	10∶0			2.4															
Lauric	12∶0				9.7														
Myristic	14∶0	3.9								3.8	6.1								
Pentadecanoic	15∶0			1.1						1.2	1.8	0.6							
Palmitic	16∶0	12.3	22.0	15.9	32.1	15.3	15.6	22.4	40.8	37.9	26.8	22.7							
Sapienic	16∶1 n−9				5.7				2.3										
Palmitoleic	16∶1 n−7	10.7	3.1	6.2	16.1	2.1	0.0	1.3	6.7	4.3	3.4	0.4							
Argaric	17∶0									4.3	2.1								
Stearic	18∶0	7.3	5.5	17.8	17.6	0.5	2.9	4.0	4.5	12.6	10.8	1.6							
Oleic	18∶1 n−9	18.4	10.7	16.1	10.6	22.7	35.2	36.5	8.2	11.1	12.5	17.3							
Vaccenic	18∶1 n−7		3.1		0.0	1.8				2.9	2.0	1.1							
Linoleic c	18∶2 n−6	22.1	30.6	5.6	3.2	33.7	43.3	30.1	15.4	2.0	3.3	50.6							
Gamma-linolenic	18∶3 n−6								8.5										
Arachidic	20∶0			3.3	5.1	1.6	0.8	0.9	3.6		1.7								
Rumelenic	18∶3 n−3		21.7	0.6		1.0	0.8	0.8				2.3							
Linoleic t	18∶2 n−6					9.4													
Gadoleic	20∶1 n−9	14.5	3.3	1.0			0.9	1.0				2.4							
Linoleic t	18∶2 n−6					10.1													
Eicosadienoic	20∶2									0.5									
Dihomo-g-linolenic	20∶3					1.8					3.6								
Behenic	22∶0						0.2	0.4											
Arachidonic	20∶4 n−6			10.2						2.7	2.4								
Eicosapentaenoic	20∶5 n−3	3.9		10.1				1.4	6.0	5.1	5.2								
Lignoceric	24∶0			0.9			0.4	1.2	0.7										
Adrenic	22∶4 n−6	3.8		5.5					3.4	1.2	5.0	0.9							
Docosahexaenoic	22∶6 n−3	3.0		3.1						10.5	13.4								
**δ^15^N**		9.1	1.9	7.8	2.8	0.4	4.0	4.3	0.5	11.9	11.5	4.4							
**δ^13^C**		−20.7	−25.7	−18.9	−21.3	−27.1	−11.9	−24.4	−37.5	−19.2	−19.2	−27.8							
**% N**		8	4	13	7	4	1	2	10	9	9	3							
**% C**		44	43	51	42	42	42	40	45	38	38	41							
**% P**		1.6	0.2	0.9	1.6	0.2	0.3	0.2	0.8	2.0	4.0	0.8							
**Mosquito Fatty acid**																			
	**AP100 ♀(3)**	**AP100 ♂(3)**	**Bean ♀(3)**	**Bean ♂(3)**	**BLP ♂(2)**	**BLP♀(3)**	**Brewer’s yeast ♀(3)**	**Brewer’s yeast ♂(3)**	**Chick Pea ♀(3)**	**Chick pea ♂(3)**	**Corn ♂(1)**	**Rice ♀(3)**	**Rice♂(2)**	**Spirulina ♀(3)**	**Spirulina ♂ (3)**	**SLP ♂ (3)**	**Tuna meal ♂(1)**	**Wheat bran ♀(3)**	**Wheat bran ♂(3)**
Caprylic																			
Lauric	0.3	0.5		0.6	0.4	0.3	0.6	0.6	0.2	0.5		0.4		0.6	0.5	0.3	0.6		
Myristic	3.7	4.6	0.5	3.3	1.4	1.2	2.9	1.9	0.7	2.0		1.1	1.1	2.2	2.1	1.8	2.2	0.4	4.7
Pentadecanoic	2.6	1.1			0.5				2.0	3.3		0.3		1.0	0.7	0.3	0.8		1.6
Palmitic	31.0	32.3	28.5	29.3	23.6	27.1	21.1	27.6	25.5	28.8	20.7	35.3	35.1	37.8	32.7	29.7	27.9	30.9	32.7
Sapienic	0.6	1.1			1.0	2.5	0.1	0.6	1.3	0.8	1.2	0.8	0.6	1.3	0.9	0.7	1.2	0.0	0.5
Palmitoleic	10.6	9.9	10.8	13.5	15.5	18.4	34.1	35.5	6.8	12.1	11.8	14.5	14.2	15.1	16.5	7.4	6.0	5.7	8.2
Argaric	0.4	0.4			0.6			0.1	0.3	0.3	0.6			0.9	1.1	2.6	1.9		
Stearic	4.8	2.6	3.7	3.7	5.4	4.5	1.9	2.0	1.4	1.4	6.6	1.6	0.9	2.8	2.8	5.7	4.4	5.3	6.0
Oleic	21.8	23.0	15.9	16.8	27.9	25.5	20.9	22.4	16.1	15.0	34.4	21.7	22.6	10.9	13.4	29.6	29.8	15.6	14.8
Vaccenic	2.3	1.2	2.1		1.8	1.6	2.9	3.1	2.5	1.7	3.8	1.3	0.0	1.1	1.1	2.2	2.5	0.8	1.1
Linoleic c	11.0	10.7	18.9	13.7	7.1	7.4	5.4	3.6	36.0	28.5	7.0	18.8	25.7	16.5	17.8	3.3	5.6	29.9	20.8
Gamma-linolenic	0.8				0.4									5.2	5.0				
Arachidic	0.2	0.2	0.7		0.8		1.2	0.7	0.2	0.2		0.2			0.3	0.4			
Rumelenic	1.0	0.9	18.4	16.3	0.7		1.2		1.8	1.7	1.2	0.2				0.4		0.6	0.6
Linoleic t	0.8	0.8			0.4	3.2			1.8	1.4	0.0	0.5			0.8	0.1		0.8	1.6
Gadoleic	0.2	0.2														0.1			
Linoleic t	0.1	0.7	0.5		0.2				2.0	1.6		0.6		1.2	1.2			2.0	1.7
Eicosadienoic				1.7	0.3		0.9		0.4	0.3		0.2							
Dihomo-g-linolenic		0.3			1.0	0.5	1.4	0.3	0.5	0.2				0.5	0.6	0.2		0.6	
Behenic																			
Arachidonic	0.9	0.2			5.8	4.6		0.3	0.2	0.2	3.3	1.7		1.7	1.8	3.2	3.0	5.2	5.7
Eicosapentaenoic	6.5	8.7			5.3	3.1	5.3	1.3	0.4	0.3	9.5	0.8		1.2	0.7	11.3	12.5	2.3	
Lignoceric																			
Adrenic																			
Docosahexaenoic	0.2	0.4														0.7	1.6		
**δ^15^N**	9.7	10.1	3.1	3.0	9.9	10.0	5.0	5.2	1.0	1.5	6.6	6.9	6.8	3.0	3.4	12.3	12.1	7.7	8.2
**δ^13^C**	−19.9	−20.4	−25.2	−25.4	−18.1	−18.2	−21.2	−21.3	−27.1	−27.5	−11.4	−25.1	25.1	35.5	−37.5	−18.6	−18.9	−26.4	−26.5
**TBN (µg N)**	38	41	29	26	37	33	34	31	33	30	20	34	31	39	24	29	28	22	22
**TBC (µg C)**	159	205	114	101	147	129	140	130	149	152	95	144	147	172	99	118	122	81	83
**TBP (µg P )**	5.8	3.4	4.7	3.3	4.8	3.5	3.9	3.2	6.8	3.6	1.8	4.4	2.1	5.7	3.6	2.9	2.9	5.9	3.1

SLP and BLP stands for Squid and Bovine liver powder respectively.

**Figure 3:**
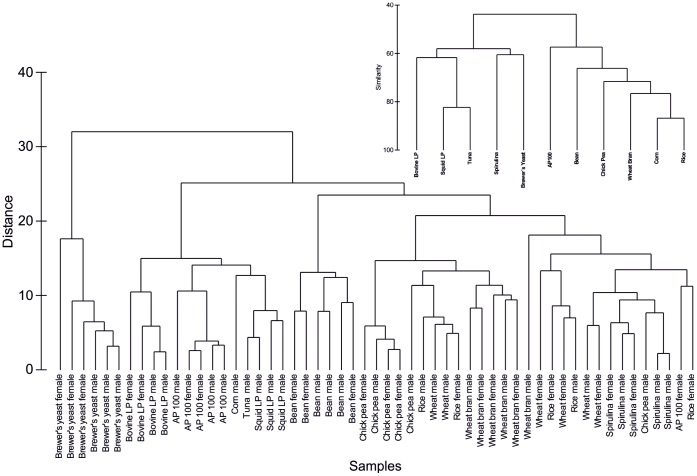
Experiment 2. Cluster analysis of fatty acid profiles of *Anopheles arabiensis* mosquitoes fed specific diets, based on Euclidean distance (insert based on Bray Curtis similarity).

The most common fatty acids present in all the diets were the palmitic (16∶0, 100% occurrence, 12–40% relative fatty acid composition (RFAC)), palmitoleic (16∶1 n7, 100% occurrence, 3–16% RFAC), stearic (18∶0, 100% occurrence, 0.5–18% RFAC), oleic (18∶1 n9, 100% occurrence, 8.2–37% RFAC) and linoleic (18∶2 n6, 100%, 2–51% RFAC) acids (Table 1). In addition, spirulina contained the characteristic gamma linolenic acid (18∶3 n6) and all leguminous samples contained the characteristic (18∶3 n3) rumelenic acid. In a matrix of larval diet versus relative fatty acid composition of diet, cluster analysis clustered the cereals closely and the fish products closely, as would be expected (Figure 3, insert).

Simply plotting RFAC of diet, against the RFAC of the resultant mosquitoes, yielded highly significant relationships; in most cases accounting for over 50% of the variability in the data, suggesting that the majority of fats are taken up and preserved indiscriminately, as was evident from the patterns observed in Figure 2. On closer analysis it became clear that the significant relationships were a reflection of the dominant fatty acid profiles of the diets skewing the data. Average relative fatty acid composition of the most common acids in the diets were palmitic (16∶0, 31.1% s.d. 22.9), palmitoleic (16∶1n7 4.5% s.d.4.6), stearic (18∶0, 7.5% s.d. 6.1), oleic (18∶1 n9, 16.5% s.d.10.2) and linoleic (18∶2 n6, 18.5% s.d. 17.7). In the mosquitoes the average dominant acids were palmitic (16∶0, 30.3% s.d. 6.1), palmitoleic (16∶1n7 14.4% s.d. 8.4), stearic (18∶0, 3.2% s.d. 2.1), oleic (18,1 n9, 20.0% s.d.5.6), linoleic (18∶2 n6, 16.4% s.d. 9.9). The diet data showed broad similarities in FA profiles, somehow reflecting the uniformity of the building blocks required for the essential structures of life; however the lower standard deviation values in the mosquitoes suggested that as consumers they also have some influence on their specific fatty acid profiles. These data also suggest there is preferential accumulation from the diet of palmitoleic and oleic FAs by the mosquitoes.

To establish whether “you are what you eat” [36], a matrix of mosquito and diet versus relative fatty acid composition was constructed. At the first level, mosquitoes of the same sex which were fed the same diet showed the highest degree of similarity, with Euclidean distances of less than 10. At the next level the mosquitoes fed the same diet showed the greatest degree of similarity, at the next level it appeared that there was some clustering based on whether the mosquitoes were fed a cereal, fish or legume diet. Notably, diet had a stronger influence than gender on the fatty acid profile of the individual mosquitoes, suggesting that there is a high degree of nutritional plasticity and providing strong evidence for dietary routing (Figure 3.) [20]. This led to the rejection of hypothesis 1 that fatty acid composition of mosquitoes is fixed and not influenced by the diet.

**Figure 4:**
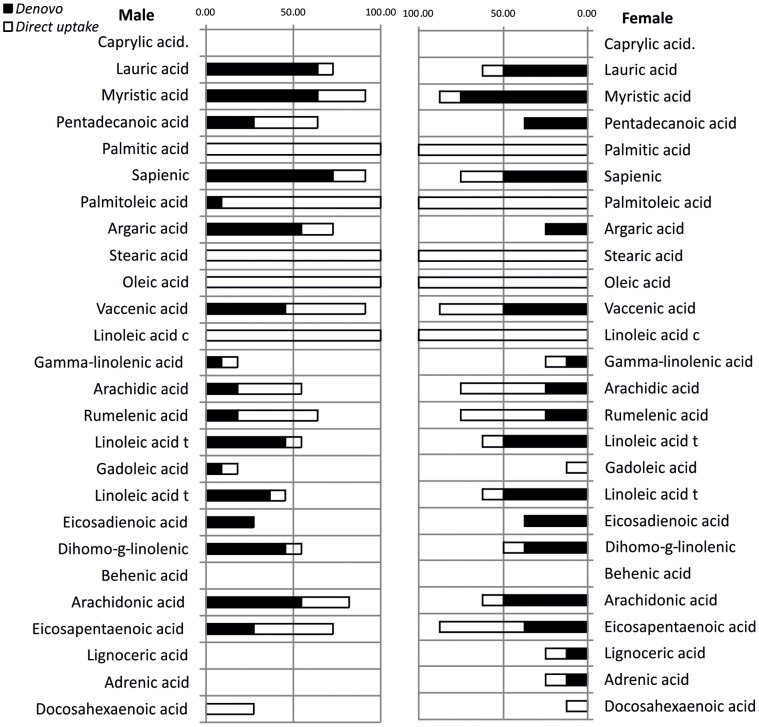
Experiment 2. Graphic showing the occurrence of *de-novo* synthesis or direct uptake as a percentage of the total population analysed. NB. Any number under 100% indicates the fatty acid was not present in all the *Anopheles arabiensis* mosquitoes analysed.

In an attempt to generate comparable information from the large data set and reveal the flow and synthesis of individual fatty acids up the food chain, graphically evident from Figure 3, in a simple mathematical manner, both the food and the mosquitoes were scored depending on the presence (1) or absence (0) of a particular fatty acid. This allowed us to compute and graphically present (Figure 4) direct uptake, *de-Novo* synthesis and frequency of occurrence of each fatty acid in all the mosquitoes fed on the range of diets presented. Presence or absence classifications based on binary systems have been widely used in medical studies and ecological modelling [37]. In this system the cut off threshold for absence was 0% RFAC and presence was deemed anything above 0% RFAC. The sum of the binary values for the mosquitoes over the sum of the binary values for the diet factorised (i.e. multiplied by the number of mosquitoes (typically n = 3) obtained and measured from that diet) was computed. If the value for mosquitoes was greater than the value of diet (factorised), they were scored with a 1 and designated *de-novo* synthesis. The sum of these values was computed for each fatty acid and calculated as a percentage of the number of diets used (to successfully rear mosquitoes) in the overall analysis (11 diets male, 8 diets female), this allowed male and female values to be compared. To determine the occurrence of direct uptake, if the sum of the binary value of the mosquitoes and the binary value of the factorised diet was greater than number of mosquitoes used in the analysis it was assumed that there was direct uptake and a value of 1 was assigned and again calculated as a percentage of the number of diets used for both males and females. This has a simple logic, as if there was no fatty acid present in the diet the factorised value of the diet would be zero, and thus only when the fatty acid was present in both the diet and the mosquito would the sum of the values be greater than the number of mosquitoes used, and thus the number 1 can be assigned to indicate direct uptake.

The fatty acids present in all the mosquitoes were the palmitic (16∶0, 100% occurrence, 12–40% RFAC), palmitoleic (16∶1 n7 100% occurrence, 3–16% RFAC), stearic (18∶0, 100% occurrence, 0.5–18% RFAC), oleic (18∶1 n9, 100% occurrence, 8.2–37% RFAC) and linoleic (18∶2 n6, 100%, 2.0–51% RFAC) acids; these are all common fatty acids found in a range of food stuffs [24], [20]. The analysis suggested that these were all directly taken up from the diet, this fits the hypothesis of dietary routing [15], which suggests that organisms will take up and use FAs in their original form to avoid energy loss or the cost associated with modification (Figure 4.).

To further examine the level of dietary routing a cluster analysis was performed on the “raw” data of the 28 possible fatty acids, by producing a matrix of each average diet RFAC versus average mosquito RFAC (n = 11 male, n = 8 female). This allowed us to estimate, on an individual fatty acid basis, the apparent transfer and conservation of RFAC profile up the food chain from a range of diets. For this analysis resemblance matrices were constructed based on Bray Curtis similarity. In female mosquitoes the highest diet to mosquito similarities were in the linoleic t (98%) linoleic c (80%), oleic (80%) palmitic (80%) and rumelenic (78%), gamma linolenic (64%) fatty acids. It could be argued that these are also the fatty acids most commonly found in both diets and the mosquitoes analysed, with only the rumelenic (25% *de-novo* synthesis, 50% direct uptake) and gamma linolenic (13% *de-novo* synthesis, 13% direct uptake) not present in all of the mosquitoes and diets analysed. In the males the highest diet to mosquito similarities were in palmitic (82%) rumelenic (78%), gamma linolenic (70%), argaric (60%) acids. The percentage occurrence of *de-novo* synthesis was similar for rumelenic (20% *de-novo*, 40% direct uptake) and gamma linolenic (10% *De-novo* 10% direct uptake) acids in both males and females. However argaric acid was exclusively produced by *de-novo* synthesis in only 30% of the females and 54% of males, and obtained by direct uptake in about 5% of males from the squid liver powder (SLP) treatment. Although females of the SLP treatment were viable and of average size based on total body carbon data, they were not successfully analysed for fatty acids due to technical problems, possibly leading to this discrepancy.

**Figure 5:**
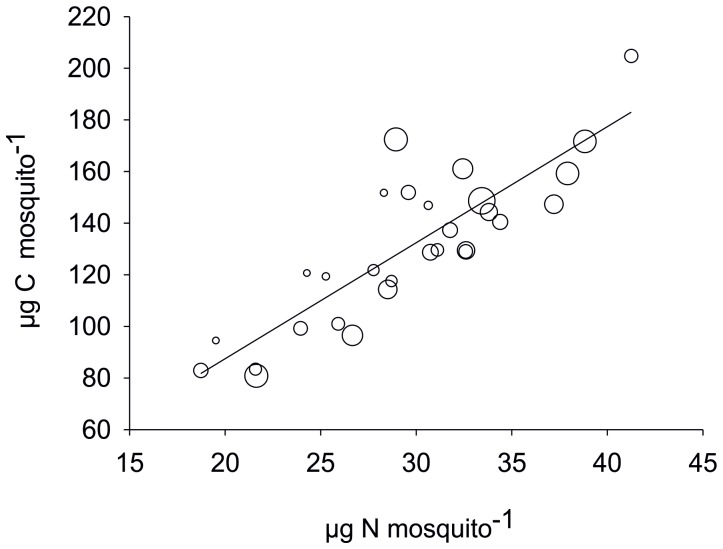
Regression analysis of average (n = 3) TBN against TBC, where bubble size represents TBP, Experiment 2.

There was a weak but highly significant correlation between the independent variables TBN and % N of the diet the mosquitoes were fed (r^2^ = 0.17, F_1,86_ = 14.22, p = 0.0003), but not with % C or % P of the diet. There were no simple correlations between TBC or TBP and elemental dietary composition. There were strong correlations and highly significant relationships between TBC and TBN for both males (R^2^ 0.794, F_1,34_ = 188.40 p = <0.0001) and females (R^2^ 0.85, F_1,32_ = 188.40 p = <0.0001) in experiment 2 (Figure 5). It could be argued that these are not independent variables, however there were no significant relationships between the TBC and TBP either, for either males or females (Figure 5). Multiple regression analysis (Table 3) revealed that 66% of the variation in the TBC of male mosquitoes could be explained by the fatty acids and stoichiometry of the diet; this was a significant interaction (p = <0.000004). For TBN, 93% of the variation could be explained by the diet (p = <0.00000) and for total body P (TBP) an astonishing 99.7% of the variation could be explained for by the dietary composition (p = <0.00000). In the females only 19% of the variation in TBC (p = <0.05), 80% of TBN variation (p = <0.00000) and a similarly high 99.8% of TBP variation (p = <0.00000) could be explained by the dietary composition.

**Table 3:** Beta values of multivariate analysis of mosquitoes against diet Experiment 2.

		Males			Females		
		µg C	µg N	µg P	µg C	µg N	µg P
	**Corrected R^2^**	0.661	0.935	0.998	0.189	0.804	0.998
	**P**	0.00000	0.00000	0.00000	0.038	0.00000	0.00000
	%C	−0.79	−0.43				
	%N		−1.9			0.69	
	%P				0.82		
10∶0	Caprylic acid						
12∶0	Lauric acid	−6.2					−0.29
14∶0	Myristic acid					1.22	
15∶0	Pentadecanoic acid						
16∶0	Palmitic acid	−0.05	0.43				0.03
16∶1 n−9	Sapienic						
16∶1 n−7	Palmitoleic acid	5.72					
17∶0	Argaric acid			−0.26			
18∶0	Stearic acid					−0.87	
18∶1 n−9	Oleic acid	0.92		−0.05	0.40	0.28	
18∶1 n−7	Vaccenic acid						
18∶2 n−6	Linoleic acid c	1.97	1.27	0.10	−0.74	−0.63	
18∶3 n−6	Gamma-inolenic acid	−1.5	−2				
20∶0	Arachidic acid						
18∶3 n−3	Rumelenic acid						
18∶2 x	Linoleic acid t		−0.6				
20∶1 n−9	Gadoleic acid		1.59				
18∶2 x	Linoleic acid t						
20∶2	Eicosadienoic acid			1.12			
20∶3	Dihomo-g-linolenic acid						1.12
22∶0	Behenic acid						
20∶4 n−6	Arachidonic acid						
20∶5 n−3	Eicosapentaenoic acid		1.66				−0.68
24∶0	Lignoceric acid						
22∶4 n−6	Adrenic acid		2.33	0.08			
22∶6 n−3	Docosahexaenoic acid		−1.6				

Regression analysis of δ^15^N diet against δ^15^N of the subsequent mosquito yielded an equation of y = 0.877×+2.495 with an r^2^ of 0.931, the overall shift being around 2.5 ‰ (Table 2). Diet to mosquito shifts in carbon isotope signatures were within the expected range, of around 1 ‰; regression analysis of δ^13^C diet against δ^13^C mosquito yielded the equation y = 1,017×+0.732 with an r^2^ = 0.991.

### Experiment 3. Determining the Influence of Dietary N and P on Mosquito Survival and Production

**Figure 6:**
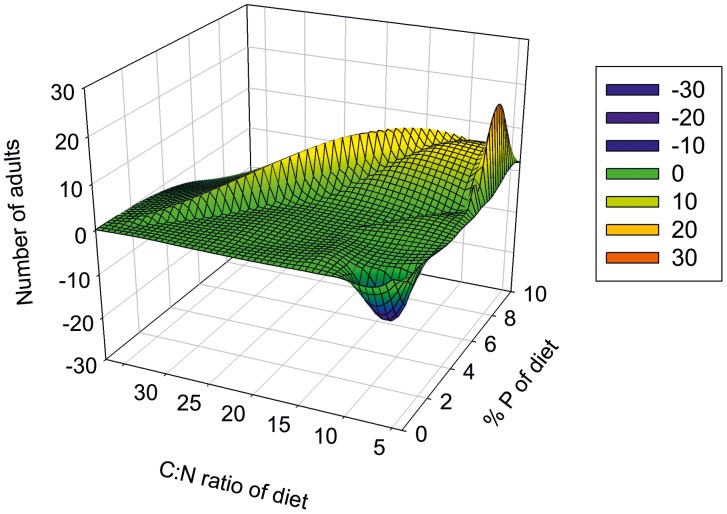
Adult production per dish (n = 3) against dietary quality, Experiment 3.

In experiment three percentage P in the diet appeared to have a greater impact on adult “production” per dish than dietary % N (Figure 6), both showing weak but highly significant correlations (p<0.00000, r^2^ = 0.40, F_1,46_ = 22.60 and p<0.0006, r^2^ = 0.23, F_1,46_ = 13.52 for P and N respectively).

## Discussion

These analyses taken together suggest that larval dietary quality and quantity have a substantial impact on adult population size, wing length, survival and possibly fecundity in *An. arabiensis* mosquitoes. We have shown that fatty acids such as arachidonic acid [32] which have previously been shown to be essential for Culex species were only present in around 80% of male and 60% of female mosquitoes, with *de-novo* synthesis evident in at least 50% of the mosquitoes sampled. The highest levels of similarity between mosquito arachidonic RFAC and diet were in steric acid in the males (40%) and eicosaptaenoic acid in the females (35%), suggesting that these acids may play a role in the synthesis of arachidonic acid in mosquitoes. In addition there were high levels of *de-novo* synthesis of arachidonic acid (>5% RFAC) in the wheat bran fed mosquitoes; wheat bran had high levels of linoleic acid (50% RFAC) which is an established precursor of arachidonic acid [38]. Arachidonic acid is rare in the plant kingdom, but it can be found in some fungi, mosses and ferns and is a major component of several microalgae, where it reaches up to 47% of the triglyceride pool [39]. This could explain the link with mosquito larval nutrition, macro-benthic algae being omnipresent in most natural larval ecosystems. Previous work has suggested that mosquitoes have to obtain the C18-22 fatty acids from their diet as they are unable to elongate the 18C acids, [40], [41], [42], [43], [44]. They have shown that eicosapentaenoic acid (EPA), docosahexaenoic acid (DHA) and arachidonic acid (AA) [40], [44], [45], [46] are all essential acids; however in these experiments there was substantial evidence of *de-novo* synthesis in the 18C plus group, in both males and females. Whether this is the result of elongation or shortening, is unclear from our results, but by using individually stable isotope labelled fatty acids and a pyrolysis system linked to an isotope ratio mass spectrometer it should be possible to further elucidate these pathways [38]. Only decohexaenoic acid (DHA) in both males and females and gadoleic acid in males, were exclusively directly routed from the diet but were not present in all the mosquitoes, suggesting they are not essential, with the caveat that these experiments did not study the full life cycle of these *An. arabiensis* mosquitoes. These fatty acids previously identified as essential for mosquitoes may be necessary to complete the full life cycle.

Whether the *de-novo* synthesis of fatty acids takes place within the mosquito or within the microbial or macro-benthic biofilm in the larval trays is unclear from these experiments, but a number of bacteria have been shown to be able to synthesise these FAs [47], and mosquitoes are known to graze on bacterial cells in the water column [41]. An attempt was made to detect this intermediate trophic level by using the isotopic data from both the food and diet. It is commonly quoted in isotopic circles that “you are what you eat plus a few per mille (‰)” [36], since there is a ubiquitous and characteristic shift in the δ^15^N signal as you move up the trophic ladder, due to the discrimination against heavier ^15^N atoms by the enzymatic and kinetic reactions. One step up the trophic ladder usually results in delta shift of 2–3 ‰, thus we hypothesised that if larval grazing of bacteria was a dominant source in the larval diet we should be able to see a characteristic shift of around 4–6% from the diet to the mosquito as it would reflect the two trophic levels. Regression analysis of δ^15^N of food against δ^15^N of subsequent mosquitoes was around 2.5 ‰, suggesting that direct food uptake rather than bacterial grazing was the dominant process. Unfortunately, published data for isotopic shifts from diet to bacteria is scarce to non-existent, despite extensive data mining, thus this result suggests either that bacterial grazing does not contribute significantly to larval nutrition or that bacterial grazing does contribute significantly, but there is no characteristic isotopic shift from substrate to product during bacterial growth. There was a weak but significant interaction (R^2^ = 0.241, F_1,68,_ p = 0.0149) between ratio of the diet and delta shift from diet to mosquito which could hint at the role of bacterial processing of diet in the higher C:N treatments, or could be a reflection of starvation which has been shown to increase diet to organism delta shifts [48]. In retrospect it would have been astute to do both isotopic and fatty acid analysis of the biofilms which are commonly present in detectable quantities on the bottom of the larval trays.

An attempt was made to determine the influence of dietary fatty acids profiles and dietary stoichiometry on mosquito stoichiometry and some measure of mosquito fitness or competitiveness (fitness in the context of sterile mosquitoes could be construed as a misnomer). Wing length as a measure of mosquito size is a well-accepted determinant of mosquito competiveness. Given that we found significant correlations between wing length and both TBC and TBN in the initial exploratory experiment and that it is logistically easier to analyse for TBN and TBC than wing length when running isotope analysis, we used TBC and TBN as a measures of mosquito competitiveness. It is important to remember that these mosquitoes were raised at a low larval density and non-limiting conditions and were sampled as tenerals and not fed as adults. In experiment 2, regression analyses of % N, C and % P in the diet versus mosquito TBN and TBC showed that only % N of diet was significant otherwise no significant interactions were observed; this suggests that % N of the diet has an influence on the TBN and thus wing length, and competiveness of the mosquito. Additionally in experiment 3, we showed that both % N and % P of the diet had a significant impact on population size. In experiment 2 multivariate analysis suggested there was a greater predictability of mosquito TBP from the dietary composition, which was clearly a result of the degree of its bioavailability and stability. Phosphorous, most probably being phospholipid derived, was therefore the link with the fatty acid profile and not lost from the system, in contrast to the nitrogen which can be complexed within lignin type substances and not readily nutritionally available [49]. In addition, excess N can be lost from the system as gas through the processes of denitrification. Oleic and linoleic acid c appeared to have most consistent influence on total body stoichiometry (Table 3), with % N and % P in the diet significantly contributing to the model of µg N and µg P in the female mosquitoes (Table 3.).

Notably, although there were strong correlations and highly significant relationships between TBC and TBN for males and females, respectively, in experiment 2, there were no significant relationships between the TBC and TBP for either males or females (Figure 5). The average C:N:P ratio of all the diets was 106∶9:1, ranging from 243∶10:1 in bean to 9∶4:1 in tuna meal, the average value for all the female mosquitoes was 28∶7:1, and for all the males was 48∶10:1. These results reveal divergent male and female stoichiometry, females having a much higher P requirement with an average of 5 µg P per female and 2.9 µg P per male (s.d 1.0 in both cases). In addition the C:N ratio of the mosquitoes was more tightly bound than their C:P ratios with %SD of the C:P ratio five times greater than the %SD of the C:N ratio. Previous research has shown that approximately 50% of the total body carbon and nitrogen of teneral mosquitoes is structural and does not turnover within the lifetime of the mosquito [26], and that these mosquitoes can accumulate up to three times their initial body carbon from sucrose solutions [15]. Results from experiment 1 and 2 therefore suggest that mosquito size is primarily determined by nitrogen availability. Experiment 3 was set up to determine the impact of diet quality or stoichiometry on production and survival akin to “mosquito production per unit food”. This was achieved by keeping dietary carbon concentrations constant across treatments and initial larval density to a minimum to overcome any negative feedbacks of overfeeding. Although % P in the diet had a greater impact on adult “production” compared to dietary % N, the stronger influence of dietary P on survival may once again have been the result of greater dietary P bio-availability. Nitrogen and phosphorous interactions are notoriously difficult to untwine and it is apparent that dietary quality has a significant influence on the production and survival of the adult mosquito, as seen in Figure 6 [50].

When we combine the evidence from all the experiments presented, it appears that mosquito size and consequent competitiveness is controlled by the nitrogen content and maybe more importantly nutritional bio-availability of that nitrogen from the larval food source. Conversely, concentration of P is often quoted as the degree of overall productivity of the aquatic system [51]. The general stoichiometric mismatch between diet and mosquitoes would suggest that nitrogen is indeed limiting both in the laboratory and the natural environment. In the tuna meal, squid liver powder, and brewer’s yeast treatments this was not the case, indicating that maybe these larvae were carbon rather than nitrogen limited. However, it is unlikely that these dietary configurations would be observed in nature. We hypothesise that in *An. arabiensis* mosquitoes nitrogen content and thus mosquito size is controlled by upper and lower limits of nitrogen cycling, the lower limit being determined by the nitrogen availability of the diet and the upper limit being posed by the fouling of the larval water due to build up of toxic ammonium products either as the result of excretion or mineralisation, the breakdown of organic nitrogen to inorganic nitrogen by the microbial communities in the larval water. Larval overfeeding often leads to high larval mortality and *An. arabiensis* is known as a clean water species [52]. Indeed additional simple chemical analysis [53] showed that larval water ammonium concentration in the healthy *arabiensis* trays was around 2 ppm NH^+^_4_ compared to 100 ppm NH^+^_4_ in the larval trays of *albopictus* species.

We hypothesise that total larval nitrogen availability linearly determines the overall size of the mosquito which ranged from 20 µg N/80 µg C to 58 µg N/261 µg C, almost a threefold difference in TBC or mosquito size, and that P is not only present as a structural component linked to specific phospholipids, but is also a more flexible storage component, evident from the data shown in Figure 5. This hypothesis would explain why there is little correlation between total body C and P values but a strong correlation between fatty acid profiles and total body P, contrary to stoichiometric theory. Therefore in essence we reject Hypothesis 2 and replace it with “Teneral individual C:N is fixed and not influenced by the larval diet”, as it appears that teneral C:N ratios are fairly fixed with values of 4.2∶1 and 4.5∶1 and % SD of less than 7 and 11% for males and females, respectively. It could be argued that stoichiometric theory is more applicable to aquatic environments but these mosquitoes were sampled as non-fed adults and as such were not subjected to a terrestrial dietary environment. Some explanation could be offered by the terrestrial feeding ecology of the mosquito: male mosquitoes notoriously only feed on sugar sources in the adult stage, which are presumably low in phosphorus.

Dietary restriction, or even having a flexible P store, could possibly improve longevity, as most aquatic systems are either P or N limited [54], [55]. This mechanism of N determining size, and P determining longevity and mosquito abundance would result in an evolutionarily successful flexible trade off strategy between size and longevity. Small mosquitoes live longer increasing probability of finding a mate, whereas larger individuals play hard, win the mate and die young, as has been shown in other insect species such as crickets [56].

Body size is often strongly correlated with fighting ability, or resource-holding potential (RHP), such that the larger of two competing males usually wins the contest [57], [58], [59]. Larger male mosquitoes have been reported to be more successful in mating than smaller ones [60], [61]. Intriguingly female body size has also an advantage in mate selection, larger females of *An. gambiae* s.s. being preferentially selected for mating [62]. Field studies have noted a positive correlation between female body size, which is presumably influenced by larval nutrition and competition, and parity status [2], [63].

On the other hand, dietary restriction in insects has generally been shown to increase longevity [64], and longevity can in turn lead to an increased chance of mating. Indeed this flexible hypothesis for mosquitoes is backed up by a very recent, as yet unpublished, study of *Anopheles gambiae* s.l. [65] in which mean adult male body size significantly influenced adult survival (F-value = 51.847; P<0.01) and correlated with larval nutrition (r = 0.946; P<0.01). Males that consumed the greatest amounts of food had the lowest survival (F-value = 4.491, P = 0.012) with a mean survival of 11 days. This data suggests that the smallest ones had the highest levels of longevity.

The lack of influence of phosphorus on overall insect size contrasts with the findings of Visanuvimol and Bertram [66] who found that P availability in the diet influenced cricket weight and size (although intriguingly not total carbon); however they also found that dietary P had little influence on cricket life span. These contradictory findings could reflect the extremely different life cycles of cricket and mosquitoes. Indeed what the authors did stress was that there was a significant relationship between total body carbon and nitrogen, but they also found that neither total body carbon nor nitrogen were correlated with total body phosphorous, thus also failing to demonstrate strict stoichiometric interactions. In line with our hypothesis they did find that older insects were more depleted in P, suggesting that P stores are used up as insects get older and are not replenished. Woods *et al*., [67] also suggested that P content may be only weakly related to body mass. They suggested that several taxa exhibit inverse dependence of P content on body size (e.g. plants).; Nielsen *et al.* [68] again supporting our nascent hypothesis.

### Conclusions

In conclusion, pyrolysis GCMS allowed a comprehensive analysis of fatty acid profiles of single mosquitoes and their diets to be undertaken which revealed the common occurrence of *de-novo* synthesis of a number of important fatty acids. It also suggested that fatty acids play an important role in the P nutrition of mosquitoes. The analysis revealed that diet has a greater influence on fatty acid profiles than gender, suggesting that dietary routing is an important mechanism in mosquitoes. *An. arabiensis* mosquitoes appear to exhibit a highly plastic feeding strategy characteristic of generalist feeders and are able to feed on a range of fatty acids and diet qualities, an ability that allows them to exploit a range of micro habitats dominated by different primary producer species. The stoichiometric-centric analysis suggested that *An. arabiensis* individual adult size is determined by the upper and lower limits of nitrogen availability and that population-size is determined by the total phosphorus availability of the system with the consequence that phosphorous is a flexible storage product of the adult mosquito. These findings are in line with new paradigms about quality/quantity issues in ecology [69], which shift away from a biomass density variable to include a two state paradigm, which represents populations or groups in a food web in terms of both their quality and quantity [69]. Given the simplicity and rapidity of the sample analysis described herein, we suggest that these methods could be useful to further test the models presented by Getz and Owen [69] on a logistically feasible scale. These results lay out an experimental foundation on which to conduct future research both in the field and laboratory and may explain why increased malaria incidences have been observed and reported in areas with higher inorganic fertiliser usage [70], [71].
